# Growth and Bulb Yield of Garlic as Influenced by Clove Size

**DOI:** 10.1155/2021/7351873

**Published:** 2021-11-05

**Authors:** Bizuayehu Desta, Netsanet Tena, Getachew Amare

**Affiliations:** ^1^Department of Horticulture, College of Agriculture and Natural Resource Sciences, Debre Berhan University, P.O. Box 445, Debre Berhan, Ethiopia; ^2^Department of Plant Sciences, College of Agriculture and Natural Resources Sciences, Debre Berhan University, P.O. Box 445, Debre Berhan, Ethiopia

## Abstract

Garlic is an important cash crop in many regions of Ethiopia. However, the yield of the crop is constrained by several factors. Among these, inappropriate clove size is one of the major agronomic practices that can decrease the yield of the crop. Hence, a field experiment was conducted during the 2020/21 main cropping season at Debre Berhan University, College of Agriculture and Natural Resource Sciences, demonstration and research site to evaluate the effect of clove size on growth and bulb yield of garlic (*Allium sativum* L.). The treatments consisted of five clove sizes: 1–1.49 g, 1.5–1.99 g, 2–2.50 g, 2.51–2.99 g, and 3–3.5 g. An improved variety “Tseday” was used as a planting material. The experiment was laid out as a randomized complete block design in a factorial arrangement and replicated for three times. The results revealed that clove size significantly influenced all growth and yield parameters of garlic. Planting of 3–3.5 g cloves reduced days to emergence by 11 and 6.33 days and days to maturity by 28.33 and 18.00 days, respectively, as compared to planting of 1–1.49 g and 2–2.5 g cloves. This treatment also increased total bulb yield by 25.88% and 15.58%, respectively, as compared to planting of 1–1.49 g and 2–2.5 g cloves. In addition, this treatment significantly increased most of the growth and bulb yield components. Hence, it can be concluded that planting of 3–3.5 g cloves could be recommended to enhance early emergence, good vegetative growth, and total bulb yield of garlic.

## 1. Introduction

Garlic (*Allium sativum* L.) belongs to Alliaceae family, and it is believed to be originated in Central Asia (India, Afghanistan, West China, and Russia) and spread to other parts of the world through trade and colonization [1, 2]. The value of garlic as a crop has been recognized from very ancient times; it is estimated that it has been cultivated for over 5000 years. It is the second most widely produced *Allium* next to onion and used as a seasoning in many foods and as condiment [3, 4]. It is a fundamental component in many dishes from various countries in the world including Ethiopia, adding taste to foods as well as it helps to make them more palatable and digestible [5]. As a cash crop, it is used to earn foreign currency by exporting to Europe, the Middle East, and African countries [6]. The oil of garlic is volatile and has sulfur-containing compounds that are responsible for the strong odor, unique flavor, and pungency as well as for health benefits [7]. Among the ten top garlic producers, China is the largest producer accounting for over 78% of world total production (22.27 million tons). Other major producing counties were India, Bangladesh, Republic of Korea, Egypt, Spain, USA, Uzbekistan, Russia, and Myanmar, respectively (FAOSTAT, 2018). Ethiopia has placed number 15 in the world ranking [8].

In 2018, the world production of garlics was 28.49 million tons from 1,546,741 hectares of land. According to FAOSTAT 2018 report, the Ethiopian total production is 124,801 tons from 12,429 hectares of land with an average productivity of 10.04 tons/ha, which is very low compared to world average 18.4 tons/ha [8]. This is due to various biotic and abiotic stresses, including low soil fertility, inappropriate clove size, planting density, irrigation schedule or shortage of rainfall, and lack of improved varieties resistant to major diseases and insects [9–11]. Among these, inappropriate clove size is one of the main factors limiting the productivity of garlic in Ethiopia [12, 13].

Several researchers have shown that the size of the cloves, which is planted, affects the size of the harvested bulb. Jones and Mann [14] and Brewster [15] reported that the relation between the size of cloves planted and the size of bulbs harvested have a linear relationship in that bulb size increased markedly as the size of planted cloves increased. Plant height was also positively correlated with the size of planted cloves. They further stated that yield increased as the size of mother bulbs was changed from small to large. This again appears to be an effect of the clove size because larger mother bulbs were produced using larger cloves of garlic. Similarly, Rubatzky and Yamaguchi [16] also described that individual clove weight of garlic greatly influenced yield potential in that large-sized cloves consistently outyielded small-sized cloves. Various studies on the other hand have reported the direct influence of seed size on growth and yield of the harvested bulb. Stahlschmidt et al. [17] reported that the biggest seeds produced more vigorous plants, with greater leaf area and with larger bulb diameters than smaller seeds. However, in Ethiopia, the use of medium-sized cloves (2–2.5 g) that was nationally recommended and widely used recorded very low yield as compared to the world and African average yield. Therefore, determining the appropriate clove size for better growth and bulb yield is critical. Hence, the objective of this study was to evaluate the effect of clove size on growth and bulb yield of garlic.

## 2. Materials and Methods

### 2.1. Description of the Experimental Site

The experiment was conducted on demonstration and research site of Debre Berhan University during the growing season of 2020/21. The university is located at 130 km away from Addis Ababa and at a latitude and longitude of 9°41′ N 39°32′E/9.683° N 39.533° E, respectively, and altitude of 2840 m.a.s.l. The rainy season of the area is characterized by a bimodal rainfall with an average rainfall of 927.10 mm. The mean maximum and minimum temperatures per month range from 18.3°C to 21.8°C and from 2.4 to 8.9°C, respectively. According to the FAO soil classification system, the most dominant soil in the area is vertisol [18].

### 2.2. Treatments and Experimental Design

The treatment consisted of five clove size categories, namely, 1–1.49 g, 1.5–1.99 g, 2–2.50 g, 2.51–2.99 g, and 3–3.5 g. At planting time, cloves were separated from the bulbs and sorted and graded according to their size category. The treatments were arranged in a randomized complete block design in a factorial arrangement with three replications.

### 2.3. Planting and Agronomic Practices

Experimental plots were ploughed, thoroughly harrowed, and leveled and ridges of about 20 cm high were prepared. The plot size was 3.6 m^2^ (1.8 m × 2 m). A distance of 1 m between the plots and 1.5 m between blocks was maintained. A garlic cultivar called “Tseday” was used for the experiment. Cloves of different sizes were planted on June 15, 2020, during the rainy season according to the standard planting density of 10 × 30 cm with 20 plants per row. Fertilizer was applied at the rate of 92 P_2_O_5_ kg ha^−1^ and 105 N kg ha^−1^ in the form of DAP and urea with equivalent amount of 200 and 150 kg ha^−1^, respectively, where all the recommended rate of DAP was applied at the time of planting to all plots uniformly. The applications of urea were splitted into one-third during planting, one-third at active vegetative growth (three weeks after plant emergence), and one-third six weeks after plant emergence, as side dressing. Weeding, chemical sprays, and harvesting were done according to the recommendation for the crop as described by Getachew and Asfaw [9].

### 2.4. Data Collected

Ten sample plants from each plot were randomly tagged from the middle four rows and data were recorded on the growth parameters of garlic. Vegetative parameters related to growth (growth parameters) were measured on the sample plants: plant height (cm), leaf length and width (cm), and shoot dry mass (g). Additionally, two extra parameters have been recorded: (i) days to 50% emergence of shoots and (ii) 75% of maturity of plants were recorded. Yield components were measured after harvest from each treatment, namely, neck diameter (cm), bulb length and diameter (cm), average bulb weight (g), and total dry biomass (g).

Total bulb yield was calculated from total plants harvested from the central four rows of each plot and expressed on a hectare basis. To determine bulb dry matter, cloves from five bulbs randomly selected were chopped into small pieces with the help of stainless steel knife and mixed thoroughly, and the exact weight of each sample was determined and recorded as fresh weight. The samples were placed in paper bags and dried in an oven at 70°C until a constant weight was obtained. Each sample was immediately weighed using digital sensitive balance and recorded as dry weight. Percentage of dry matter content for each sample was calculated by the following formula:

(1) $$\begin{array}{c} \text{DW}=\frac{\left[\left(\text{DW}+\text{CW}\right)-\text{CW}\right]}{\left[\left(\text{FW}+\text{CW}\right)-\text{CW}\right]}\times 100, \end{array}$$

where DW = dry weight, CW = container weight, and FW = fresh weight.

### 2.5. Data Analysis

Data obtained were subjected to analysis of variance (ANOVA) using the General Linear Model (GLM) of the SAS statistical package version 9.2. All significant pairs of treatment means were compared using Duncan's multiple range test (DMRT) at 5% level of significance.

## 3. Results and Discussion

### 3.1. Phenological Parameters

#### 3.1.1. Days to Emergence

Days to emergence was significantly (*P* < 0.001) affected by clove size (Table 1). Planting of 3–3.5 g cloves has shortened days to emergence by 11 and 6.33 days, respectively, as compared to planting of 1–1.49 g and 2–2.5 g cloves. The earlier emergence of garlic plants from the large-sized cloves than small-sized cloves could be attributed to the availability of higher amounts of stored nutrients to provide an optimal supply of carbohydrate for the emerging seedlings of the larger cloves. This result was in agreement with the findings of Ahmed et al. [19] and Castellanos et al. [20] who reported that large-sized cloves emerged earlier than small-sized cloves. In addition, Abdulkadir [13] also stated that the longest duration for emergence was required by plants from the small-sized cloves whereas the shortest duration was required by plants from large-sized cloves.

#### 3.1.2. Days to Maturity

Clove size significantly (*P* < 0.001) influenced days to maturity (Table 1). Planting of 3–3.5 g cloves has shortened days to maturity by 28.33 and 18 days, respectively, as compared to planting of 1–1.49 g and 2–2.5 g cloves. This could be due to the higher carbohydrate and mineral reserves in large-sized cloves that led to earlier establishment and in turn resulted in earlier maturity. According to this finding, Lencha and Buke [21] reported that the higher carbohydrate and mineral reserves in large bulbs produce vigorous plants that establish faster and complete its maturity earlier when compared to those from smaller bulbs. On the contrary, Abdulkadir [13] and Nasir et al. [22] described that garlic clove sizes did not significantly influenced days to maturity. This could be due to the genetically controlled character of days to maturity in garlic that could not be altered by the use of different clove sizes.

### 3.2. Growth Parameters

#### 3.2.1. Plant Height

Plant height was significantly (*P* < 0.001) affected by clove size (Table 2). The longest plant height (74.40 cm) was recorded by planting of 3–3.5 g cloves, which was statistically at par with 2.51–2.99 g cloves. However, the shortest plant height (63.80 cm) was recorded by planting of 1–1.49 g cloves, which was statistically similar to planting of 1.5–1.99 g cloves. The longest plant height due to planting of large-sized cloves might be ascribed to more reserve nutrients present in the clove, which produces vigorous plants that attributed to the formation of longer plant [23]. These results agree with Kotagariwar et al. [24] who also reported that larger sized cloves resulted in increased plant height due to more reserve of nutrients in the clove during initial stages of growth in garlic. Rahim et al. [25] also stated that the plant height declined as the size of mother bulb was reduced. In contrast to this study, Gedamu [12] and Abdulkadir [13] described that clove sizes did not significantly affect plant height of garlic. This could be due to the genetically controlled character of plant height in garlic that could not be altered by the use of different clove sizes.

#### 3.2.2. Leaf Length

Leaf length was significantly (*P* < 0.01) affected by clove size (Table 2). The longest leaf length (56.36 cm) was recorded by planting of 3–3.5 g, which was statistically at par with 2.51–2.99 g cloves. However, the shortest leaf length (49.56 cm) was recorded by planting of 1–1.49 g cloves, which was statistically similar with planting of 1.5–1.99 g cloves. The longest leaf length due to planting of large-sized cloves might be ascribed to having higher nutrient reserves in the large-sized cloves that enable to provide energy more rapidly to the growing seedlings, which produce vigorous plants and in then this led to the formation of longer leaf. This result was in agreement with the finding of Ahmed et al. [19] who reported that availability of more food reserves in cloves allowed young garlic plants to be more vigorous in their growth and development. Similarly, Danna et al. [26] stated that large-sized cloves produced garlic plants that were taller. In addition, Hossain et al. [27] described that planting of large cloves resulted in the highest leaf length. On the other hand, Gedamu [12] described that clove sizes did not significantly affect leaf length of garlic. This could be due to the genetically controlled character of leaf length in garlic that could not be altered by the use of different clove sizes.

#### 3.2.3. Leaf Width

Leaf width was significantly (*P* < 0.001) affected by clove size (Table 2). The widest leaf width (2.55 cm) was recorded by planting of 3–3.5 g cloves. However, the narrowest leaf width (1.85 cm) was recorded by planting of 1–1.4 g cloves, which was statistically similar with planting of 1.5–1.99 g cloves. The widest leaf width due to planting of large-sized cloves might be ascribed to the higher nutrient reserves of large-sized cloves that enhanced the production of vigorously growing plants that led to the formation of wider leaves. This result was in agreement with the finding of Ahmed et al. [19] who reported that availability of more nutrient reserves in cloves allowed young garlic plants to be more vigorous in their growth and development. On the contrary, Gedamu [12] and Abdulkadir [13] described that clove sizes did not significantly affect leaf width of garlic. This could be due to the genetically controlled character of leaf width in garlic that could not be altered by the use of different clove sizes.

#### 3.2.4. Leaf Number

Clove size was significantly (*P* < 0.01) affected by leaf number (Table 2). The highest leaf number (12.73) was recorded by planting of 3–3.5 g cloves, which was statistically at par with 2.51–2.99 g cloves. However, the lowest leaf number (11.66) was recorded by planting of 1–1.49 g cloves, which was statistically similar with planting of 1.5–1.99 g cloves. The highest leaf number due to planting of large-sized cloves might be ascribed to the comparatively more reserve nutrients present in the larger-sized cloves, which allows vigorous growth that enable greater number of leaves. Similarly, Memane et al. [28] reported that the increased clove weight resulted in increased number of leaves in garlic. Danna et al. [26] also reported that large-sized cloves produced plants that were taller and had more leaves per plant. In addition, Baten et al. [29] and Mahmud [30] also reported that the large clove size was superior to medium and small seed cloves in number of leaves. On the contrary, Gedamu [12] and Abdulkadir [13] described that garlic clove sizes did not significantly affect the number of leaves. This could be due to the genetically controlled character of leaf number in garlic that could not be altered by the use of different clove sizes.

#### 3.2.5. Shoot Dry Mass

Shoot dry mass was significantly (*P* < 0.01) affected by clove size (Table 3). The highest shoot dry mass (5.66 g) was recorded by planting of 3–3.5 g cloves, which was statistically at par with 2.51–2.99 g cloves. However, the lowest shoot dry mass (5.10 g) was recorded by planting of 1–1.49 g cloves, which was statistically similar with planting of 1.5–1.99 g cloves. The highest shoot dry mass due to planting of large-sized cloves might be ascribed to the higher availability of stored nutrients in the large-sized cloves that resulted in luxurious vegetative growth (higher plant height, leaf length, leaf width, and number of leaves) that led to enhanced assimilates production. This ultimately caused a higher shoot dry weight. In agreement with this, Baten et al. [29] and Mahmud [30] reported that the large-sized cloves were superior to medium- and small-sized cloves in weight of leaves.

#### 3.2.6. Neck Diameter

Neck diameter was significantly (*P* < 0.001) affected by clove size (Table 3). The widest neck diameter (1.153 cm) was recorded by planting of 3–3.5 g cloves. However, the narrowest neck diameter (1.026 cm) was recorded by planting of 1–1.4 g cloves, which was statistically similar with planting of 1.5–1.99 g cloves. The widest neck diameter due to planting of large-sized cloves might be ascribed to the higher availability of stored nutrients in the large-sized cloves that resulted in maximum number of leaves that produced maximum food materials, which were stored in bulb that resulted in the maximum diameter of garlic neck. This result agrees with Ara [31] who stated that large-sized cloves resulted in maximum diameter of garlic neck.

### 3.3. Yield and Yield Components

#### 3.3.1. Bulb Length

Bulb length was significantly (*P* < 0.001) affected by clove size (Table 3). The longest bulb length (4.03 cm) was recorded by planting of 3–3.5 g cloves. However, the shortest bulb length (3.45 cm) was recorded by planting of 1–1.49 cloves, which was statistically similar with planting of 1.5–1.99 g cloves. The longest bulb length due to planting of large-sized cloves might be ascribed to the higher availability of stored nutrients in the large-sized cloves that resulted in the higher leaf area index, which leads to a higher accumulation of photoassimilates and translocation to bulbs. This, in turn, caused an increase in bulb length. In line with this, Lencha and Buke [21] described that the higher carbohydrate and mineral reserves in large bulbs produce vigorous plants that establish faster and have better development when compared to those from smaller bulbs and in turn caused a higher accumulation of assimilates and translocation to bulbs. Similarly, Mahadeen [32] reported that the seed cloves with lower weights resulted in a reduced bulb length.

#### 3.3.2. Bulb Diameter

Bulb diameter was significantly (*P* < 0.001) affected by clove size (Table 3). The widest bulb diameter (4.39 cm) was recorded by planting of 3–3.5 g cloves. However, the narrowest bulb diameter (3.54 cm) was recorded by planting of 1–1.4 g cloves, which was statistically similar with planting of 1.5–1.99 g cloves. The widest bulb diameter due to planting of large-sized cloves might be ascribed to the higher availability of stored nutrients in the large-sized cloves that resulted in the higher leaf area index, which leads to a higher accumulation of photoassimilates and translocation to bulbs. This, in turn, caused an increase in bulb diameter [20]. Similarly, Lencha and Buke [21] reported that the higher carbohydrate and mineral reserves in large bulbs produce vigorous plants that establish faster and have better development when compared to those from smaller bulbs and in turn caused a higher accumulation of assimilates and translocation to bulbs. In addition, Mahadeen [32] reported that the seed cloves with lower weights resulted in a reduced bulb diameter.

#### 3.3.3. Clove Number per Bulb

Clove number was significantly (*P* < 0.001) affected by clove size (Table 3). The highest clove number (13.45) was recorded by planting of 3–3.5 g cloves. However, the lowest clove number (10.65) was recorded by planting of 1–1.49 g cloves, which was statistically similar with 1.5–1.99 g cloves. The increase in clove number per bulb due to planting of large-sized cloves could be ascribed to the higher amount of reserve nutrients in large-sized cloves, which promoted vigorous vegetative growth, and increased production of assimilates and translocation to the cloves. This ultimately resulted in larger clove weight that caused larger clove number per bulb. Similarly, Ahmed [19] obtained that clove size had significant effect on clove number per bulb and it increased as the clove size increased. In contrast to this finding, Gedamu [12] described that clove sizes did not significantly affect clove number per bulb of garlic. This might be due to the higher amount of stored nutrients in large-sized cloves could be responsible for the increased average clove weight by increasing clove length and diameter rather than the increased in clove number per bulb.

#### 3.3.4. Average Clove Weight

Average clove weight was significantly (*P* < 0.01) affected by clove size (Table 3). The highest average clove weight (3.42 g) was recorded by planting of 3–3.5 g cloves. However, the lowest average clove weight (2.76 g) was recorded by planting of 1–1.49 g cloves which was statistically similar with 1.5–1.99 g cloves. The highest average clove weight due to planting of large-sized cloves might be due to the higher stored nutrients in large-sized cloves that produced vigorous vegetative growth and increased production of assimilates and translocation to the cloves which help for the development of larger clove length and diameter. This ultimately caused a larger average clove weight. The present study was in agreement with the findings of Adekpe et al. [33] who indicated that garlic plants which attained higher vegetative growth early possibly had developed larger clove length and clove diameter which ultimately increased the average clove weight.

#### 3.3.5. Average Bulb Weight

Clove size significantly (*P* < 0.001) affected average bulb weight (Table 4). The highest average bulb weight (36.11 g) was recorded by planting of 3–3.5 g cloves. However, the lowest average bulb weight (27.77 g) was recorded by planting of 1–1.49 g cloves which was statistically similar with planting of 1.5–1.99 g cloves. The highest average bulb weight due to planting of large-sized cloves might be ascribed to the higher amount of reserve nutrients in large-sized cloves that showed early emergence and vigorous plant growth and produced maximum number of leaves that consequently resulted in increased photosynthesis and production of assimilates and translocation to bulbs. This ultimately resulted in the development of larger bulbs. In agreement with this, Rahman and Das [34] reported that the highest bulb weight was obtained by planting of larger cloves. In addition, Rahim et al. [25], Bhuiya et al. [35], and Adekpe et al. [33] indicated that garlic plants which attained higher vegetative growth early possibly had developed larger bulbs.

#### 3.3.6. Bulb Dry Matter Content

The percentage of dry matter content was significantly (*P* < 0.001) affected by clove size (Table 4). The highest value (32.53%) was recorded by planting of 3–3.5 g cloves and the lowest dry matter content was recorded by planting of 1–1.49 g cloves which was statistically similar with planting of 1.5–1.99 g cloves. The highest percent dry matter content due to planting of large-sized cloves might be due to the higher amount of stored nutrients in large-sized cloves that enhanced vigorous growth and development of plant and ultimately, resulting in higher accumulation of photo assimilates (dry matter) and translocation to the bulbs. Similarly, Hossain et al. [27] reported that planting of large-sized cloves resulted in the highest total dry matter content. On the contrary, Gedamu [12] described that clove sizes did not significantly affect bulb dry matter content of garlic. This might be due to the fact that larger-sized cloves have comparatively more moisture content which results in higher physiological losses and ultimately lower percentage of dry matter content.

#### 3.3.7. Total Dry Biomass

Total dry biomass was significantly (*P* < 0.001) affected by clove size (Table 4). The highest total dry biomass (21.68 g) was recorded by planting of 3–3.5 g cloves. However, the lowest total dry biomass (18.04 g) was recorded by planting of 1–1.49 g cloves. The highest total dry biomass due to planting of large-sized cloves might be ascribed to the higher amount of reserve nutrients in large-sized cloves that resulted in vigorous vegetative growth of plants like leaf number, leaf diameter, leaf length, plant height, and root weight that contribute for improved rate of photosynthesis and assimilate production in the vegetative part and partitioning to the bulbs. This in turn resulted in a higher total dry biomass. The present studies are congruent with Memane et al. [28] who reported that increased vegetative and bulb growth observed in large-sized cloves due to more reserve nutrients that in turn might increase the overall total dry biomass of garlic.

#### 3.3.8. Total Bulb Yield

Total bulb yield was significantly (*P* < 0.001) affected by clove size (Table 4). The highest total bulb yield (13.13 t/ha) was recorded by planting of 3–3.5 g cloves. However, the lowest total bulb yield (10.43 t/ha) was recorded by planting of 1–1.49 g cloves which was statistically similar with planting of 1.5–1.99 g cloves. The highest total bulb yield due to planting of large-sized cloves could be due to the higher amount of stored nutrients in large-sized cloves that resulted in the increased formation of vegetative structure for nutrient absorption that led to increased photosynthesis and production of assimilates to fill the sink and in then increased bulb size and weight. Similarly, Memane et al. [28] reported that increased vegetative and bulb growth observed in large-sized cloves due to more reserve nutrients that in turn might increase the overall yield of garlic. In addition, other researchers, Adekpe et al. [33], Bhuiya et al. [35], and Rahim et al. [25], indicated that garlic plants which attained higher vegetative growth early possibly had developed larger bulbs and higher yield. Ara [31] also stated that the highest yield was found from large-sized cloves while the small-sized cloves gave the lowest yield.

## 4. Conclusion

Our findings suggest that garlic growth and yield are directly related to the size of cloves planted. Therefore, planting cloves of the appropriate size can improve garlic production. Clove size showed significant effects on all growth and yield parameters of garlic. The results of the present study indicated that planting of 3–3.5 g cloves significantly reduced days to emergence and maturity and provide the highest yield of garlic. Hence, it can be tentatively recommended that production of the highest yield of garlic is achieved by using 3–3.5 g cloves. As a result, incomes of smallholder garlic producers could be significantly improved if the recommended clove size is applied to garlic crops cultivated in the study area.

## Figures and Tables

**Table 1:** Effect of clove sizes on days to emergence and maturity.

Clove sizes (g)	Days to emergence	Days to maturity
1–1.49	22.00^a^	168.66^a^
1.5–1.99	20.66^a^	165.66^a^
2–2.5	17.33^b^	158.33^b^
2.51–2.99	12.00^c^	144.66^c^
3–3.5	11.00^c^	140.33^d^
CV (%)	6.07	1.14
Level of significance	^*∗∗∗*^	^*∗∗∗*^

Means within a column followed by the same letters are not significantly different at the prescribed level of significance. ^*∗∗∗*^Significant at 0.1% probability level.

**Table 2:** Effect of clove sizes on plant height, leaf length, width, and number.

Clove sizes (g)	Plant height (cm)	Leaf length (cm)	Leaf width (cm)	Leaf number
1–1.49	63.80^d^	49.56^d^	1.85^d^	11.66^d^
1.5–1.99	66.46^cd^	51.40^cd^	2.02^cd^	11.86^cd^
2–2.5	68.73^bc^	53.08^bc^	2.19^bc^	12.26^bc^
2.51–2.99	71.43^ab^	54.13^ab^	2.32^b^	12.50^ab^
3–3.5	74.40^a^	56.36^a^	2.55^a^	12.73^a^
CV (%)	2.55	2.59	4.42	1.93
Level of significance	^*∗∗∗*^	^*∗∗*^	^*∗∗∗*^	^*∗∗*^

Means within a column followed by the same letters are not significantly different at the prescribed level of significance. ^*∗∗*^ and ^*∗∗∗*^indicate significance at 1% and 0.1% probability level, respectively.

**Table 3:** Shoot dry mass, neck diameter, bulb length and diameter, clove number per bulb, and average clove weight of garlic as influenced by the effect of clove sizes.

Clove sizes (g)	Shoot dry mass (g)	Neck diameter (cm)	Bulb length (cm)	Bulb diameter (cm)	Clove number per bulb	Average clove weight (g)
1–1.49	5.10^d^	1.026^d^	3.45^d^	3.54^d^	10.65^d^	2.76^c^
1.5–1.99	5.23^cd^	1.046^cd^	3.56^cd^	3.61^cd^	11.16^d^	2.85^c^
2–2.5	5.36^bc^	1.073^bc^	3.67^bc^	3.87^bc^	11.74^c^	3.04^b^
2.51–2.99	5.56^ab^	1.096^b^	3.79^b^	3.96^b^	12.48^b^	3.14^b^
3–3.5	5.66^a^	1.153^a^	4.03^a^	4.39^a^	13.45^a^	3.42^a^
CV (%)	2.13	1.32	2.04	3.80	2.55	3.18
Level of significance	^*∗∗*^	^*∗∗∗*^	^*∗∗∗*^	^*∗∗∗*^	^*∗∗∗*^	^*∗∗*^

Means within a column followed by the same letters are not significantly different at the prescribed level of significance. ^*∗∗*^ and ^*∗∗∗*^ indicate significance at 1% and 0.1% probability level, respectively.

**Table 4:** Average bulb weight, total bulb yield, bulb dry matter content, and total dry biomass as influenced by the effect of clove sizes.

Clove sizes (g)	Average bulb weight (g/plant)	Total bulb yield (t/ha)	Bulb dry matter content (%)	Total dry biomass (g)
1–1.49	27.77^d^	10.43^d^	26.13^d^	18.04^d^
1.5–1.99	28.55^cd^	10.73^cd^	27.20^cd^	18.85^c^
2–2.5	30.44^bc^	11.36^bc^	28.26^bc^	19.43^c^
2.51–2.99	31.33^b^	11.96^b^	29.33^b^	20.23^b^
3–3.5	36.11^a^	13.13^a^	32.53^a^	21.68^a^
CV (%)	4.24	3.17	2.87	2.00
Level of significance	^*∗∗∗*^	^*∗∗∗*^	^*∗∗∗*^	^*∗∗∗*^

Means within a column followed by the same letters are not significantly different at the prescribed level of significance. ^*∗∗∗*^Significant at 0.1% probability level.

## Data Availability

Primary data were used to support this study.
